# Editorial: Harnessing Neuroplasticity in the Injured Central Nervous System Using Spinal Neuromodulation

**DOI:** 10.3389/fresc.2022.841014

**Published:** 2022-04-27

**Authors:** Cristina L. Sadowsky, Dimitry G. Sayenko

**Affiliations:** ^1^International Center for Spinal Cord Injury, Kennedy Krieger Institute, Baltimore, MD, United States; ^2^Physical Medicine and Rehabilitation, Johns Hopkins School of Medicine, Baltimore, MD, United States; ^3^Center for Neuroregeneration, Department of Neurosurgery, Houston Methodist Research Institute, Houston, TX, United States

**Keywords:** neuroplasticity, spinal cord, neuromodulation, rehabilitation, transcutaneous electrical spinal stimulation

The capacity of the central nervous system (CNS) to transform during development and adapt following a neural injury is, as of yet, not fully explored or quantified. We do know that functional connectivity is activity dependent, and that “neurons that fire together wire together” (1). We also know that quantifiable improvements in motor, sensory, and autonomic functions after spinal cord injury (SCI) can be achieved by harnessing several neuroplastic interventions, among which neuromodulation of the spinal cord via epidural or transcutaneous electrical spinal stimulation is gaining a prominent role due to its clinical effectiveness and translatability (2). A review paper by Flores et al. focuses on recent advances using animal models to study the mechanisms and effects of rehabilitation and spinal neuromodulation to restore sensorimotor function after SCI. The review addresses both lumbar and cervical stimulation and fills in translational gaps between basic and clinical sciences. Martin's mini review succinctly summarizes what has been studied in the past half a decade about functional neurorestoration following transcutaneous spinal stimulation in humans with neurologic deficits due to complete and incomplete SCI. The 13 fully reviewed papers (out of 115 screened abstracts) speak to feasibility and in large successful application of transcutaneous spinal stimulation in restoring meaningful upper and lower limbs function, using defined stimulation parameters (long pulse duration of 0.5 to 1 ms, and frequencies of 30 to 50 Hz) in a relatively safe and participant-friendly manner. Tefertiller's et al. presents pilot findings from seven individuals with complete and incomplete SCI, who underwent a prospectively applied combinatorial rehabilitative approach (transcutaneous spinal stimulation + massed and task-specific practice) to improve neuromotor function of both upper and lower limbs. As the studies with spinal neuromodulation are gaining the attention of clinicians, researchers, and the community, the cumulated knowledge and quality of this research are increasing, providing critical insights on the type and characteristics of interventions and the dose-response effects to promote a given function. Nevertheless, better reporting of recruitment methods and intervention protocols, and quantification of the observed findings must be addressed in future studies. Current tools are still lacking in the ability to *quantify* clinician assistance during activity-based training with and without spinal stimulation. The work by Linde et al. and the Mayo Clinic team sought to add on this knowledge of objective measurements and determine the validity of force sensitive resistors to quantify clinician assistance and participant progression during body weight supported treadmill training with epidural spinal stimulation in individuals with SCI. The investigators were able to observe reduced contact time by the clinicians that was related to increased stance duration, which objectively identified independence in stepping with the use of spinal stimulation-enabled stepping following chronic sensorimotor complete SCI. The above works are particularly relevant because they challenge the current view on what is inaccurately called “functional plateauing” and document concrete, specific interventions that can be immediately applied to obtain and quantify clinically and physiologically significant changes in function even long time after the neurological injury onset. Although, the neurophysiological mechanisms of spinal neuromodulation are yet to be unveiled, from a bioengineering perspective, the ultimate goal of the technological interference should be to match the intrinsic functional design of the sensorimotor networks. Use of spinal stimulation in combination with other neuromodulation approaches to further *harness* neuroplasticity on supraspinal and spinal levels of the neuroaxis, will be the following step in this exciting endeavor.

## Author Contributions

Both authors listed have made a substantial, direct, and intellectual contribution to the work and approved it for publication.

## Conflict of Interest

The authors declare that the research was conducted in the absence of any commercial or financial relationships that could be construed as a potential conflict of interest.

## Publisher's Note

All claims expressed in this article are solely those of the authors and do not necessarily represent those of their affiliated organizations, or those of the publisher, the editors and the reviewers. Any product that may be evaluated in this article, or claim that may be made by its manufacturer, is not guaranteed or endorsed by the publisher.
